# Levels of knowledge about asthma of parents of asthmatic children

**DOI:** 10.1590/S1679-45082018AO4204

**Published:** 2018-05-29

**Authors:** Cristian Roncada, Thiago de Araujo Cardoso, Bianca Martininghi Bugança, Luísa Carolina Bischoff, Karina Soldera, Paulo Márcio Pitrez

**Affiliations:** 1 Faculdade de Educação Física, Centro Universitário da Serra Gaúcha, Caxias do Sul, RS, Brazil; 2 Núcleo de Educação em Saúde da Criança, Centro Infant, Pontifícia Universidade Católica do Rio Grande do Sul, Porto Alegre, RS, Brazil; 3 Faculdade de Biomedicina, Centro Universitário da Serra Gaúcha, Caxias do Sul, RS, Brazil

**Keywords:** Knowledge, Asthma, Parents, Pulmonary medicine, Education, Public health, Conhecimento, Asma, Pais, Pneumologia, Educação, Saúde pública

## Abstract

**Objective:**

To evaluate the levels of knowledge about asthma of parents of school children.

**Methods:**

A cross-sectional study was carried out with parents of children with medical diagnosis of asthma (mild, moderate and severe), followed up at an outpatient referral center for childhood asthma in the Southern region of Brazil (Asthma Group). Parents of children with asthma in remission and healthy children were also selected (Control Group). The Newcastle Asthma Knowledge Questionnaire (NAKQ) questionnaire was applied in both groups.

**Results:**

A total of 154 parents of children participated in the study; in that, 62 (40.26%) in the Asthma Group, and 92 (59.74%) in the Control Group, with a mean age of 35.60±10.03 years. Of these, 132 (85.7%) were female, and 72 (46.8%) parents studied up to high school. The average score of level of knowledge was 18.06±4.11 points. Only 30.5% parents had acceptable levels of knowledge about asthma, which were more prevalent in the Asthma Group than in the Control Group (41.9% *versus* 22.8%, p=0.01). The mean score in Newcastle Asthma Knowledge Questionnaire (NAKQ) was higher in the Asthma Group (19.32±3.92 *versus* 17.21±4.03, p=0.001), respectively. The parents with mild and moderate asthmatic children scored more than those of severe asthma (19.5 and 19.9 *versus* 18.2 points, p=0.02).

**Conclusion:**

Most parents had an unsatisfactory level of knowledge about asthma, which reinforces the need for changes in public asthma management programs.

## RESUMO

**Objetivo:**

Avaliar os níveis de conhecimento em asma de pais de crianças escolares.

**Métodos:**

Foi realizado um estudo transversal, com pais de crianças com diagnóstico médico de asma (leve, moderada e grave), em acompanhamento ambulatorial em um centro de referência em asma infantil na Região Sul do Brasil (Grupo Asma). Foram também selecionados pais de crianças com asma em remissão e crianças saudáveis (Grupo Controle). Aplicou-se o questionário *Newcastle Asthma Knowledge Questionnaire* (NAKQ) em ambos os grupos.

**Resultados:**

Participaram do estudo 154 pais de crianças, sendo 62 (40,26%) do Grupo Asma e 92 (59,74%) do Grupo Controle, com média de idade de 35,60±10,03 anos. Destes, 132 (85,7%) eram do sexo feminino e 72 (46,8%) eram pais com escolaridade até o Ensino Médio. A pontuação média do nível de conhecimento foi de 18,06±4,11 pontos. Apenas 30,5% dos pais apresentaram níveis aceitáveis de conhecimento em asma, os quais, por sua vez, foram mais prevalentes no Grupo Asma do que no Grupo Controle (41,9% *versus* 22,8%; p=0,01). E a média de pontuação no *Newcastle Asthma Knowledge Questionnaire* (NAKQ) foi maior no Grupo Asma (19,32±3,92 *versus* 17,21±4,03, p=0,001), respectivamente. Os pais de crianças com asma leve e moderada acertaram mais do que os de asma grave (19,5 e 19,9 *versus* 18,2 pontos; p=0,02).

**Conclusão:**

A maioria dos pais apresentou nível insatisfatório de conhecimento sobre a asma, o que reforça a necessidade de mudanças nos programas públicos de manejo de asma.

## INTRODUCTION

Asthma is a chronic inflammatory disease of the airways that requires adequate management to remain controlled, avoiding exacerbations and loss of lung function.^(^ ^1^ ^)^ In Brazil, about 20 million people suffer from the disease, and the pediatric population is the most affected, reaching a prevalence of 28% in some regions.^(^ ^2^ ^)^

Lack of treatment and low compliance with medications are the greatest challenges in managing the disease, since these increase the risks of exacerbations leading to emergency departments visits, hospital admissions, and consequently, to high costs to the public services, in addition to significantly lowering the quality of life of those involved.^(^ ^3^ ^)^

Guidelines and consensus increasingly prioritize the correct usage of medications and health education of patients and their parents.^(^ ^4^ ^)^ However, health education, especially in developing countries, is not yet appropriate for the management of asthma.^(^ ^5^ ^)^

Popular myths amplify the fears about the side effects of the medications, limit engagement in physical exercise, and lead to behaviors that hinder the control of the disease.^(^ ^6^ ^)^ It is common, *e.g.*, for patients to quit using them, or for parents not giving the metered-dose inhalers prescribed by physicians, alleging heart damage and patient dependence.^(^ ^7^ ^)^ These paradigms should be overcome, but in order for this to happen, much effort is required from all involved with the disease, including healthcare professionals, patients and parents.

This challenge is believed to be greater in developing countries. In Brazil, the population's low level of literacy can interfere with treatment compliance, considering that 4% of Brazilians over the age of 15 years are totally illiterate, and 65% are between rudimentary and elementary levels of literacy.^(^ ^8^ ^)^

In many countries, prior studies demonstrated that the majority of parents of asthmatic children do not have satisfactory levels of knowledge about asthma,^(^ ^9^ ^–^ ^11^ ^)^ even though these are fundamental for disease management. In Brazil, the results are similar,^(^ ^12^ ^,^ ^13^ ^)^ consisting in one of the causes of poor control of the disease.

We believe that this study can expand data on knowledge about asthma of parents of asthmatic children in the Southern Region of Brazil, helping create and improve new methods in health education, aiming to increase compliance with treatment and provide better quality of life to patients and families.

## OBJECTIVE

To evaluate the levels of knowledge about asthma of parents of asthmatic children.

## METHODS

A cross-sectional study carried out between April 2015 and March 2016. Three distinct populations of parents of children and adolescents aged 6 to 17 years were recruited from the public schools of the region and a pediatric pulmonology outpatient clinic, at a reference center in the Southern Region of Brazil. The first population consisted of parents of children with a medical diagnosis of asthma; the second, of parents of children who never had a diagnosis of asthma; and the third, of parents of children with asthma in remission. Children with asthma were those diagnosed with the disease and with medical follow-up for at least 6 months; in treatment with control drugs; and with regular visits at one to three-month intervals. Children with asthma in remission were those with a diagnosis of asthma who did not use medication to control the disease and had not, in the previous 12 months, presented with wheezing, sleep affected by wheezing, wheezing after exercise and/or dry cough at night without having a cold, as per criteria of the International Study of Asthma and Allergies in Childhood (ISAAC).^(^ ^1^ ^)^ The diagnosis, as well as the classification of asthma severity (mild, moderate, and severe), was based on the criteria of the international guideline of the Global Initiative for Asthma (GINA).^(^ ^14^ ^)^

Children and adolescents with cognitive and motor limitations or with other chronic diseases that could compromise the evaluation of disease control were excluded. Illiterate parents could not be enrolled.

Parents of children diagnosed with asthma formed the Asthma Group; parents of children with asthma in remission or without a diagnosis of asthma made up the Control Group. Subjects from the Asthma Group were selected in the waiting room of the outpatient clinic of *Hospital São Lucas* - *Pontifícia Universidade Católica do Rio Grande do Sul,* in Porto Alegre (RS), on the day scheduled for the regular medical visit with the pediatric pulmonologist, using a direct approach of the interviewer, with explanation of the study and signatures of the Informed Consent and/or Assent Forms so that, after this, the tests could be applied. In the Control Group, the subjects were selected in the public schools of the region.

The Newcastle Asthma Knowledge Questionnaire (NAKQ) was used to assess the levels of knowledge of the disease. This is a specific instrument, developed by Fitzclarence et al.,^(^ ^15^ ^)^ in Australia, and validated by Cidade et al.,^(^ ^16^ ^)^ for the Brazilian population (Portuguese-BR). The instrument has 31 items, with 24 questions with “true” or “false” answers, and six open-ended questions. The correct answers receive a score of 1 and the incorrect, zero, with a minimal score of knowledge equal to zero and the maximal, of 31. The cut-off point of the instrument is ≥21 points for satisfactory levels (adequate) and less than 21 points for unsatisfactory levels (inadequate).

The data were collected on a table in the database Microsoft Access, version 2013 (Microsoft Corporation, Redmond, Washington, United States), and exported to the software Statistical Package for Social Science, version 20 for Windows (New York, United States).

The χ^2^ test was applied to compare the categorical data; the independent *t* test, to compare the means; and Spearman's correlation for variables of schooling level and the total score of knowledge level using NAKQ, with statistical difference stipulated at p<0.05. Categorical data are presented by absolute and relative frequencies, and the continuous values, by means and standard deviation. For comparison between the groups, the values were analyzed using the χ^2^ test and the one-way variance test (ANOVA) with Bonferroni's post hoc, adopting a significance value of p<0.05.

To calculate the sample, based on a prior Brazilian study,^(^ ^17^ ^)^ with the objective of evaluating knowledge about asthma of parents of asthmatic children admitted to a specialized service, and adopting a confidence level of 95% and sample error of 5%, a sample equal to or greater than 61 parents for the Asthma Group and 61 parents for the Control Group was needed to assess the proposed outcome.

The study was approved by the Research Ethics Committee of the *Pontifícia Universidade Católica do Rio Grande do Sul* (PUC-RS), under the consolidated opinion 73583, CAAE: 09088513.1.0000.5336. All participants agreed to take part in the study by means of an Informed Consent Form (ICF).

## RESULTS

The sample was composed of 154 parents of school-aged children, in that, 62 (40.26%) were in the Asthma Group and 92 (59.74%) in the Control Group, with a mean age of 35.60±10.03 years. Of these, 132 (85.7%) were females and 72 (46.8%) had schooling up to High School.

The mean age of patients of the Asthma Group was 10.22±3.12 years, and 33 (53.22%) were females. In this group, 23 (37.1%) children presented with mild persistent asthma, 24 (38.7%) with moderate persistent asthma, and 15 (24.2%) with severe persistent asthma.

In the Control Group, the mean age was 10.44±4.01 years, in which 59 (64.1%) were healthy, and 33 (35.9%) presented with asthma in remission.

The comparison between groups showed no difference as to age, sex, and schooling of the parents (p=0.547; p=0.055; p=0.071, respectively). In the comparison between the Asthma and Control Groups, there was no difference in the correlation between schooling level of parents and the total score relative to knowledge about asthma (r^2^: 0.175 and 0.061). The characteristics of the subjects are presented on table 1.

**Table 1:** Characteristics of 154 parents, divided by groups

Characteristic		Asthma Group (n=62)		Control Group (n=92)		p value
		n (%)	M±SD	n (%)	M±SD	
Parents						
Age			36.19±9.22		35.20±10.57	0.547
Female		57 (91.9)		75 (81.5)		0.055
Schooling						
	Elementary school	9 (14.5)		8 (8.7)		
	Junior school	12 (19.4)		21 (22.8)		0.071
	High School	36 (58.1)		52 (56.5)		
	Further education	5 (8.1)		11 (12.0)		

M: mean; SD: standard deviation. Student *t* test was used for continuous variables and χ^2^ for categorical variables.

The analysis, per question, of the number of parents who responded correctly demonstrated that the acceptable levels of knowledge were higher in the Asthma Group (41.9%) *versus* the Control Group (22.8%; p=0.01) (Table 2).

**Table 2:** Evaluation of the total and percentage of parents who matched each item, between the Asthma Group and Control Group

Item	Answers	Asthma Group n=62 n (%)	Control Group n=92 n (%)	p value
1	Cough, wheezing and shortness of breath or dyspnea	27 (43.5)	30 (32.6)	0.110
2	True	50 (80.6)	65 (70.7)	0.110
3	True	54 (87.1)	78 (84.8)	0.440
4	False	35 (56.5)	75 (81.5)	0.001*
5	False	52 (83.9)	37 (40.2)	<0.001*
6	Allergy, cold and exercise	55 (88.7)	70 (76.1)	0.037*
7	True	42 (67.7)	64 (69.6)	0.470
8	True	50 (80.6)	63 (68.5)	0.070
9	False	24 (38.7)	25 (27.2)	0.090
10	Write down the names of two types of treatments (medications) that should be regularly taken to prevent asthma exacerbations: inhaled corticosteroids, leukotriene-receptor antagonist (montelukast), long-acting beta-2 adrenergic agonists	44 (71.0)	38 (41.3)	<0.001*
11	Write down the names of three treatments (medications) that are useful during asthma exacerbation: short-acting beta-2 adrenergic agonists, ipratropium bromide or oral corticosteroids	50 (80.6)	42 (45.7)	<0.001*
12	False	27 (43.5)	26 (28.3)	0.0370
13	False	43 (69.4)	42 (45.7)	0.003*
14	False	47 (75.8)	82 (89.1)	0.025
15	False	31 (50.0)	41 (44.6)	0.3
16	False	35 (56.5)	43 (46.7)	0.1
17	False	60 (96.8)	89 (96.7)	0.68
18	True	15 (24.2)	23 (25.0)	0.53
19	False	30 (48.4)	35 (38.0)	0.13
20	False	24 (38.7)	45 (48.9)	0.13
21	A 5-year old child has an asthma exacerbation and uses 2 puffs of inhaled salbutamol (inhaler). Five minutes later, the patient is not getting better. Write down some possible reasons for what happened: insufficient dose, very severe exacerbation, poor inhaling technique or empty device	14 (22.6)	20 (21.7)	0.52
22	False	14 (22.6)	13 (14.1)	0.120
23	Write down ways that help preventing asthma exacerbation during exercises: short-acting beta-2 adrenergic agonists before exercise, improve asthma control (appropriate drug prophylaxis) or physical warming up	13 (21.0)	22 (23.9)	0.410
24	False	33 (53.2)	57 (62.0)	0.180
25	False	36 (58.1)	63 (68.5)	0.120
26	True	57 (91.9)	83 (90.2)	0.470
27	True	55 (88.7)	81 (88.0)	0.550
28	False	22 (35.5)	35 (38.0)	0.440
29	True	48 (77.4)	68 (73.9)	0.380
30	False	52 (83.9)	55 (59.8)	0.001*
31	True	59 (95.2)	73 (79.3)	0.004*
Total of acceptable levels#	≥21	26 (41.9)	21 (22.8)	0.010*

# Total number of parents that achieved acceptable levels of knowledge, considering the total score of the tool (cut-off point ≥21 points).

* p<0.05. The χ^2^ test was used to compare categorical variables between the Asthma and Control Groups.

**Figure 1:**
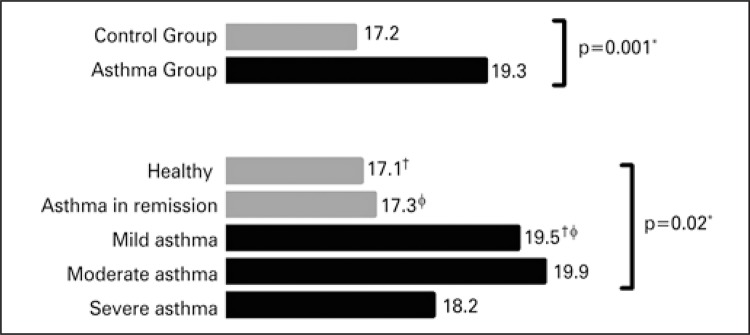
Mean scores of groups and subgroups *p<0.05;^†^ healthy *versus* moderate asthma; ^φ^ asthma in remission *versus* moderate asthma.

In the evaluation of levels of knowledge about asthma, the general mean of the sample was 18.06±4.11 points, from the maximum of 31 points stipulated by NAKQ; when analyzed by groups, the general means of correct answers were 17.21±4.03 points for the Control Group and 19.32±3.92 for the Asthma Group, (p=0.001). Only 47 (30.5%) parents showed acceptable levels of knowledge about asthma (≥21 points). The comparison among the mean scores of the subgroups showed a statistical difference only between the healthy subjects *versus* moderate asthma, and asthma in remission *versus* moderate asthma (p=0.02) (Figure 1).

**Figure 2:**
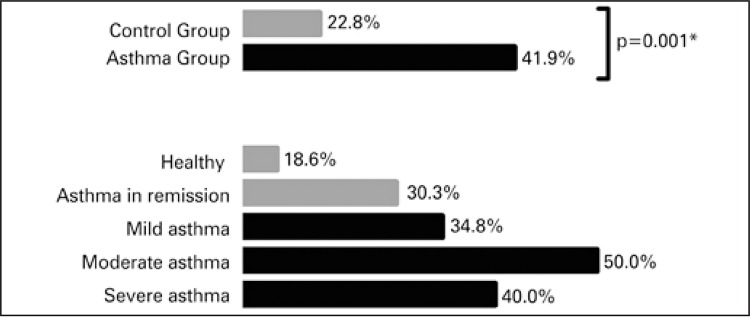
Evaluation of levels of knowledge about asthma (acceptable levels) among groups and subgroups * p<0.05.

Whereas regarding understanding of asthma, there was statistical difference only in the comparison between the groups (22.8% *versus* 41.9%), but there was no difference between the subgroups (Figure 2).

## DISCUSSION

Health education is fundamental for the adequate management of asthma. Exacerbations can be prevented, when parents of children with asthma have broader knowledge about the disease, how to identify the symptoms and triggering factors, and the actions necessary between exacerbations.^(^ ^17^ ^)^

Parents are the main link between the physician and the pediatric patient, and should understand the basic concepts of asthma. Nevertheless, in this study, the parents assessed showed lower than satisfactory levels of knowledge about asthma. To avoid biases, a correlation test was done between the level of knowledge and the schooling of parents, demonstrating that there is no such correlation.

According to NAKQ,^(^ ^16^ ^)^ we verified that 69.5% of participants did not present with satisfactory levels of knowledge about asthma, obtaining scores below 21 points. A study conducted in the Canary Islands, Spain, with 95 parents of asthmatic children seen at an emergency center, showed that approximately 80% of these parents did not know how to identify the main triggering factors of asthma exacerbations.^(^ ^18^ ^)^A study carried out in Santander, in Spain, applied to 344 parents of asthmatic children in a hospital, presented with a mean number of correct answers in the NAKQ questionnaire of 18.5 points, again showing an unsatisfactory level of knowledge.^(^ ^19^ ^)^ The similarity among the results presented in both Spanish studies and our investigation suggests that the lack of comprehension of the parents is a reality in different populations. Educational interventions, though simple, can be just as or more beneficial for the treatment of the disease as advanced and sophisticated techniques, reinforcing the need for actions of this type.^(^ ^20^ ^)^

Parents who do not have asthmatic children tend to know less about the disease. It is natural that this should occur and the results from the questionnaire be close to unsatisfactory levels, as demonstrated in this study, in which the Asthma Group had significantly more understanding about the disease than the Control Group (19.32 *versus* 17.21 points; p=0.001).

Daily living with an asthmatic individual naturally enables greater learning on the part of parents. Other studies, also conducted with parents of asthmatic and non-asthmatic children, showed similar results, indicating that the contact with asthmatic children increases knowledge about the disease.^(^ ^15^ ^,^ ^21^ ^)^ Parents of asthmatic individuals receive frequent orientations, especially due to the constant visits to outpatient clinics and emergency rooms. The results between the two groups, albeit expected, could present with different outcomes. One null hypothesis could reinforce even more the aspects related to enhanced knowledge resulting from education in asthma programs, strengthening the strong need in all populations.^(^ ^12^ ^)^

Although our results are equal to those of other studies,^(^ ^15^ ^,^ ^21^ ^)^ they are still insufficient for reaching satisfactory levels of understanding of the disease, both in the Asthma and Control Groups. This demonstrates that, even those who systematically experience the disease and receive orientation from specialized teams, do not fully master the subject entirely, evidencing the scarcity in educational approaches and increasing visits to emergency rooms.^(^ ^22^ ^,^ ^23^ ^)^

We also established that only 38.7% of parents of asthmatics answered question 20 correctly, affirming that the use of inhaled bronchodilator (salbutamol) does not damage the heart. Popular myths about health aspects are frequent, hindering comprehension and acceptance of medical treatments on the part of the parents, and leading to a decreased control of the child's asthma.^(^ ^24^ ^)^ A study also carried out with 251 parents in Southern Brazil, showed that popular myths as to childhood asthma are frequent, and that parents of asthmatic children believe more in these myths than parents of non-asthmatic children. Of the parents of asthmatic children, 29% affirmed the pressurized inhaler would cause harm to the heart, while 21% of parents of non-asthmatic children said the same (p=0.009).^(^ ^25^ ^)^ The influence of popular health myths, particularly those related to childhood asthma, is another important aspect in the management of the disease that should be better studied.

Parents of children with severe asthma tend to seek more information about the disease, because the greater the severity, the greater the need for care. Since their children are continuously symptomatic, they experience and learn about the management and treatment of the disease, which reflects in the results of studies such as ours, in which parents of children with mild and moderate asthma reached a greater score (19.5 and 19.9 points, respectively) than parents of children with severe asthma (18.2 points; p=0.02). A Brazilian study conducted with 400 children diagnosed with asthma demonstrated that (ongoing) preventive medications are underused, and oral corticoids are prescribed to try to control the disease.^(^ ^26^ ^)^ On the other hand, the North American study about the concept of severe asthma reported on the heterogeneity of the disease and the various types of treatments and medications utilized to reach adequate control, suggesting that these factors can hamper the understanding of asthma, since there are frequent changes in treatment protocols.^(^ ^27^ ^)^

The limitations of our study refer to the reduced size of the sample, bearing in mind the application in patients (Asthma Group) from a single service (especially in the quantity of patients with severe asthma), besides the use of a foreign questionnaire to evaluate knowledge about asthma − despite having been validated to Portuguese.^(^ ^16^ ^)^ Cultural aspects can influence the form of asking questions about knowledge of a specific disease. However, the authors believe that the use of a recognized and validated questionnaire increases the power to call attention to the world to this large gap in medical care, which is health education.

Considering that Brazil has an elevated prevalence of asthma in the childhood population and that the lack of control of the disease negatively impacts public health,^(^ ^28^ ^)^ programs for control of asthma, with free provision of the medication, as well as medical, psychological, and social care, with well-trained professionals, are fundamental for the effective diagnosis and treatment.^(^ ^29^ ^)^Further, the basis of these strategies should prioritize health education for parents and patients, seeking to enhance knowledge about asthma and improve the quality of life of both, thus reducing the impact of the disease on society.^(^ ^30^ ^)^

## CONCLUSION

The knowledge of parents of asthmatic children regarding asthma was insufficient, reinforcing the importance of a greater emphasis on effective education strategies about the disease.
